# Seasonal distribution and faunistic of ticks in the Alashtar county (Lorestan Province), Iran

**DOI:** 10.11604/pamj.2017.27.284.10341

**Published:** 2017-08-22

**Authors:** Behroz Davari, Firoz Nazari Alam, Hassan Nasirian, Mansour Nazari, Mohammad Abdigoudarzi, Aref Salehzadeh

**Affiliations:** 1 Department of Medical Entomology, School of Medicine, Hamadan University of Medical Sciences, Hamadan, Iran; 2 Department of Medical Entomology and Vector Control, School of Public Health, Tehran University of Medical Sciences, Tehran, Iran; 3 Reference Lab For tick Study, Department of Parasitology, Razi Vaccine and Serum Research Institute, Agricultural Research, Agricultural Research, Education and Extension Organization (AREEO) Tehran, Iran

**Keywords:** CCHF, faunistic, hard tick, soft tick, seasonal distribution

## Abstract

**Introduction:**

Ticks are non-permanent obligate parasites that have considerable medical-veterinary and zoonosis importance. In this regard a study designed to investigate the distribution and fauna of ticks in the Alashtar county in Iran from April and March 2014.

**Methods:**

Ticks were collected from livestock farms and facilities from selected rural and geographically location in the Alashtar county. Based morphological characteristics and reference identification keys, ticks were identified.

**Results:**

A total of 549 ticks including 411 hard and 138 soft ticks were found. Ten tick species including *Haemaphysalis concinna* (0.36%), *Haemaphysalis sulcata* (0.36%), *Hyalomma anatolicum* (0.18%), *Hyalomma dromedarii* (0.18%), *Hyalomma marginatum* (1.45 %), *Hyalomma schulzei* (0.36%), *Rhipicephalus annulatus* (0.18%), *Rhipicephalus bursa* (28.1%), *Rhipicephalus sanguineus* (43.63%) and *Argas persicus* (25.2%) were identified. Tick seasonal distribution were 47.26%, 22.63%, 14.96% and 15.15% in the spring, summer, autumn and winter, respectively. The tick distribution was more from plain areas (64.96%) than the mountainous areas (35.04%). The rates of the tick contamination were 97.3% and 2.7% in the traditional and industrial livestock's, respectively. The livestock contamination ranks to the hard ticks were cattle (39.51%), sheep (34.15%) and goats (26.34 %), respectively. Chi-square analysis showed a significant difference among the seasonal distribution of the ticks in the spring, summer and autumn or winter; between the tick distribution in the plain and mountainous areas; and between the traditional and industrial livestock's tick contamination (P < 0.05).

**Conclusion:**

Present study proves to change the traditional livestock's to the industrial livestock's. These findings highlight the importance of ticks and shows need to their control and tick pest management.

## Introduction

Ticks are non-permanent obligate and the most frequent ectoparasites (ticks, mites, lice and fleas) of the terrestrial vertebrates that have considerable medical-veterinary and zoonosis importance [1, 2]. They are a serious threat to animal health and public health in many parts of the world because they are able to direct damages and transmission of parasitic, viral and bacterial pathogens [3, 4]. Control of ticks and tick-borne diseases are the most important sanitation to protect livestock health and their products, particularly prevention of tick-borne diseases transmitted to humans [5, 6]. Premature and adult stages of ticks are attacked to different groups of animals and feed on their blood and interstitial fluids. They irritate their host by relatively long-term feeding and continuous saliva injection to their body. Tick feeding period lasts form several minutes in soft ticks to weeks in hard ticks that in addition to release them from one place to another, cause local discomfort of the hosts and facilitate the transmission of pathogens to the other hosts [7]. Ticks are remained infected for lifetime by feeding on their host infected blood and transfer the infection in a way of trans-ovarial or trans-stadial transmissions. Ticks also have one or more various hosts in their life cycle that they transmit the infections from wild to domestic hosts by their feeding [8, 9]. Because of any tick species may have ability to transfer a certain type of a disease. Identifying tick species, their abundance and distribution, have a great effect to understand the epidemiology of the diseases and their control in each region or globally. As much research is also being done on a vaccine against the diseases such as development of anti-tick vaccines [10], tick species identifying is also important of this aspect in the areas.

The Crimean-Congo hemorrhagic fever (CCHF) has led to an increase in the number of studies about tick distribution and tick-borne diseases [11]. Some various tick studies were done in different parts of Iran such as NorthWestern (West Azerbaijan and Ardebil Provinces), North (Guilan, Mazandaran, Golestan and Qazvin provinces), West (Kordistan and Kohgilouyeh provinces), South (Hormozgan province), Southwest (Sistan) and central [10, 12–21]. The Alashtar County with the various peripheral villages is considered one of the most important areas in terms of animal husbandry and it is the most densely populated livestock areas (nearly 250 thousand light and heavy head of cattle) in the Lorestan province from western Iran. Ticks are one of the most important pests in the livestock industry and they have the ability to transfer various diseases. Due to climate and tick life condition change during different seasons and years, it seems necessary to study the tick distribution and fauna during intermittent periods. Alashtar County has a climate and weather diversity that is livestock and animal husbandry are commonly widespread and nomadic tribes travelled there especially among the neighbor cities such as Malayer and Nahavand. In recent years, tick-borne disease cases such as Relapsing and CCHF have been reported (local reports) in the Alashtar County. Therefore, it needs to conduct a research to obtain the further epidemiological aspects about diseases caused by the ticks in the Alashtar region. So far, there is not a comprehensive study about distribution and fauna of ticks in this county. In this regard a study designed to investigate the distribution and fauna of ticks in different parts of the Alashtar county in the Lorestan province in western Iran from April and March 2014.

## Methods

**Figure 1:**
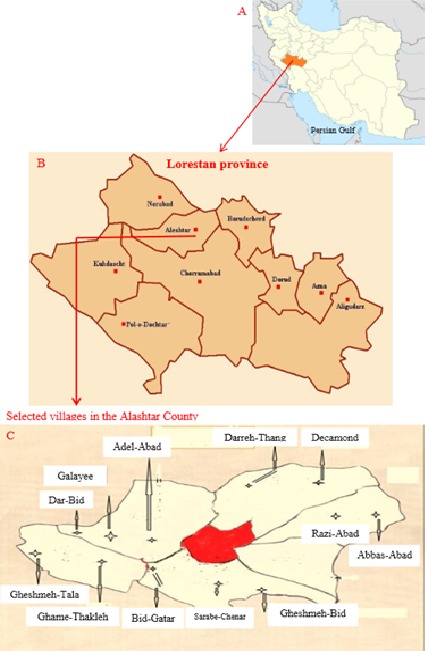
Study site maps: (A) location of the Lorestan province in Iran; (B) location of the Alashtar county in the Lorestan province and (C) location of the study areas

**Site of study**: Alashtar county is located at an altitude of 1,600 meters above the sea level in the northern of Lorestan province (Table 1, Figure 1). Nomadic herders are common among the Alashtar county peoples. Lorestan province is located in the west of Iran (Figure 1). It is a very beautiful place with a high perspiration rate and a good climatic condition, making this location green and cool and it is the route from the south to the north of Iran. This province has some popular tourist attractions with historical and cultural significance. Between the higher ranges lie many fertile plains and low hilly, well-watered districts. Lorestan covers an area of 28,392 km². The Lorestan population was estimated at 1,754,243 people in 2011. Khoramabad is the capital of Lorestan province. The major cities in this province are: Khorramabad, Borujerd, Aligoodarz, Dorood, Koohdasht, Azna, Alashtar, Noor Abad and Pol-e-Dokhtar (Figure 1). The terrain consists chiefly of mountains, with numerous ranges, part of the Zagros chain, running northwest to southeast. The climate is generally sub-humid continental with winter precipitation, a lot of which falls as snow. The average annual precipitation is 530 mm. Temperatures vary widely with the seasons and between day and night. In summer, temperatures typically range from a minimum of 12°C to a hot maximum of 32°C. In winter, they range from a minimum of -2°C to a chilly maximum of 8°C.

**Table 1:** The district and selected villages from different rural and geographically location of the Alashtar County, and livestock farms and facilities information

District	Village	Altitude (m)	Cattle				Sheep				Goat			
			Total	Con*	Cont**		Total	Con*	Cont**		Total	Con*	Cont **	
					No.	%			No.	%			No.	%
Central	Darreh-Thang	1712	280	41	3	7.32	1300	145	8	5.52	700	75	11	14.67
	Decamond	1701	310	20	2	10.0	4002	208	13	6.25	1700	83	10	12.05
	Razi-Abad	1701	312	20	6	30.0	1210	99	9	9.09	600	40	6	15.0
	Abbas-Abad	1650	320	40	8	20.0	1000	83	14	16.87	750	50	9	18.0
	Bid-Gatar	1623	180	21	3	14.29	260	15	7	46.67	198	12	6	50.0
	Gheshmeh-Bid	1521	204	31	4	12.90	1100	85	5	5.88	590	48	13	27.08
	Ghame-Thakleh	1400	117	35	4	11.43	1200	88	8	9.09	500	31	7	22.58
	Sarabe-Chenar	1516	80	13	0	0	683	45	1	2.22	240	11	1	9.09
Firoz-Abad	Adel-Abad	1600	240	30	5	16.67	1500	160	9	5.63	1500	168	8	4.76
	Dar-Bid	1650	311	45	3	6.67	840	95	12	12.63	700	59	6	10.17
	Gheshmeh-Tala	1340	280	46	7	15.22	1300	170	16	9.41	620	48	10	20.83
	Galayee	1472	410	53	2	3.77	970	159	8	5.03	580	88	5	5.68

* Con=considered livestock

** Cont=contaminated livestock

**How to select the study areas**: We divided the Alashtar county to four areas: North, South, East and West including 23, 41, 17 and 38 villages, respectively. Then we randomly selected ten percent of villages from each area. Afterward, we selected ten percent of livestock's in the each village. Subsequently, we selected ten percent of animals in the each livestock. The villages were Darreh-Thang and Decamond from North; Sarabe-Chenar, Ghame-Thakleh, Bid-Gatar and Gheshmeh-Bid from South; Razi-Abad and Abbas-Abad from East; and Galayee, Adel-Abad, Dar-Bid and Gheshmeh-Tala from West (Table 1, Figure 1).

**Figure 2:**
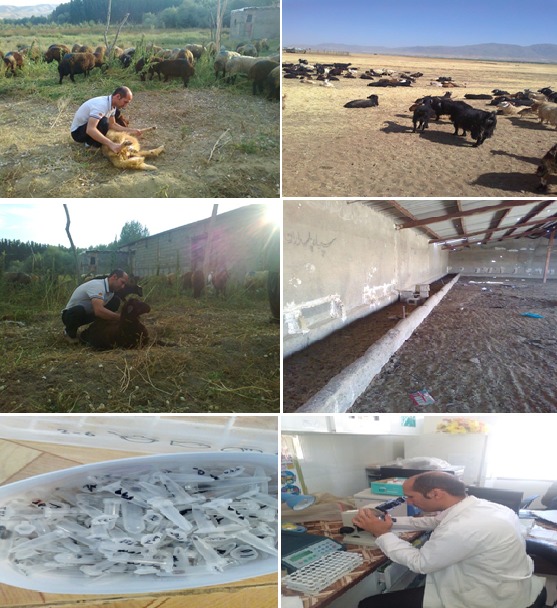
Tick collection at livestock farms and facilities in the Alashtar county

**Tick collection and identification**: The distribution and fauna of ticks were monthly studied by a cross-sectional study in different parts of the Alashtar county from April and March 2014. A total of 12 villages which had 410 households, afterward 41 livestock and subsequent 205 head of cattle for the presence of ticks were selected and studied. Ticks were collected from livestock farms and facilities from different rural and geographically location in the Alashtar county (Table 1 and Figure 1, Figure 2). Ticks were carefully separated from livestock by a curve forceps and safe anesthetic compounds such as ether and chloroform. The hard ticks were collected with taking the anterior part of the ticks at the junction to the host then they were rotated around the forceps axis or flipped and they were carefully withdrawn directly from the skin. We considered around the animal nests and crevices of livestock walls for the soft ticks [22]. The samples were placed into special pipes (Eppendorf tubes). They were immediately transferred to Medical Entomology Laboratory at School of Medicine, Hamadan University of Medical Sciences after recording detailed specifications including site collection, livestock facilities and type of animal, date of collection and environment temperature and packing. Then we followed next steps with personal protection and safe work (Figure 2). Based morphological characteristics and reference identification keys, ticks were identified to the genus, species and gender (Figure 2). For final tick identification approval, the identified samples were sent to reference acarology laboratory at the Razi Institute in Karaj.

## Results

**Figure 3:**
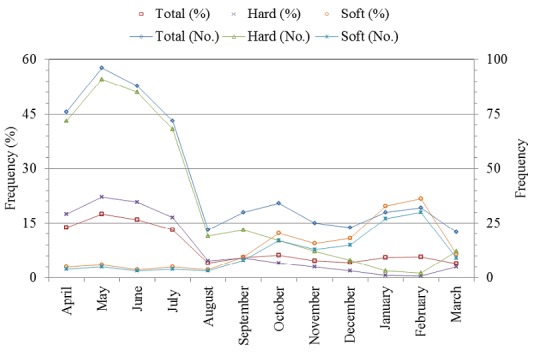
Distribution of the ticks from April to March 2014 in the Alashtar county

During twelfth monthly sampling and among the 12 selected villages in the Alashtar county, we visited 41 livestock and 205 head of cattle for the presence of ticks in the 144 round villages viewing. In fact we examined the 2460 animals at the 492 round livestock farms and facilities viewing (Table 1). A total of 549 ticks including 411 hard and 138 soft ticks were collected and identified. The highest and the lowest frequency of ticks were observed at the Abbas-Abad (15.66%) and Sarabe-Chenar (4.0%), respectively (Table 2). Figure 3 shows the frequency of the collected ticks in the Alashtar county from April and March 20^14^. The highest (17.5%) and the lowest (3.8%) frequency of ticks were observed in May and March, respectively (Figure 3). Seasonal distribution of the ticks were 47.26%, 22.63%, 14.96% and 15.15% in the spring, summer, autumn and winter, respectively (Table 3). Chi-square analysis showed a significant difference among the seasonal distribution of the ticks in the spring, summer and autumn or winter (P < 0.05) while it didn't showed a significant difference between the autumn and winter. The hard tick frequency deceased from spring to winter in the year while increased for the soft ticks (Table 3). The most frequencies of ticks were observed in the May (17.5%) and then in the June (16.0%) (Figure 3). The most tick activities were related to *Rhipicephalus sanguineus* among the seasons except *Rhipicephalus bursa* that the most activities were in the autumn. While the other species had lower activity among the spring, summer, autumn and winter (Table 3). Unlike the hard ticks, the most activities of the soft ticks were observed in the winter and then in the autumn while they had lower activities in the spring and summer (Table 3 and Figure 3).

**Table 2:** Frequency of the ticks in the Alashtar county from April to March 2014

District	Village	Tick					
		Total		Hard		Soft	
		No.	%	No.	%	No.	%
Central	Darreh-Thang	32	5.8	25	6.08	7	5.07
	Decamond	45	8.19	33	8.02	12	8.7
	Razi-Abad	75	13.66	53	12.9	22	15.94
	Abbas-Abad	86	15.66	56	13.62	30	21.74
	Bid-Gatar	35	6.37	27	6.57	8	5.8
	Gheshmeh-Bid	41	7.46	34	8.27	7	5.07
	Ghame-Thakleh	61	11.1	52	12.65	9	6.52
	Sarabe-Chenar	22	4.0	2	0.49	20	14.49
Firoz-Abad	Adel-Abad	37	6.73	28	6.81	9	6.52
	Dar-Bid	42	7.65	37	9.0	5	3.62
	Gheshmeh-Tala	48	8.74	45	10.95	3	2.17
	Galayee	25	4.55	19	4.62	6	4.35

**Table 3:** Distribution of the tick species from spring to winter 2014 in the Alashtar county

Species	Spring		Summer		Autumn		Winter	
	No.	%	No.	%	No.	%	No.	%
*Rhipicephalus sanguineus*	136	56.9	73	30.5	17	7.1	13	5.4
*Rhipicephalus bursa*	95	61.7	36	23.4	19	12.3	4	2.6
*Hyalomma marginatum*	7	87.5	0	0	1	12.5	0	0
*Hyalomma dromedarii*	1	100	0	0	0	0	0	0
*Hyalomma schulzei*	2	100	0	0	0	0	0	0
*Haemaphysalis sulcata*	2	100	0	0	0	0	0	0
*Hyalomma anatolicum*	1	100	0	0	0	0	0	0
*Rhipicephalus annulatus*	1	100	0	0	0	0	0	0
*Haemaphysalis concinna*	2	100	0	0	0	0	0	0
*Argas persicus*	12	8.7	15	10.9	45	32.6	66	47.8
Total	259	47.26	124	22.63	82	14.96	83	15.15

**Figure 4:**
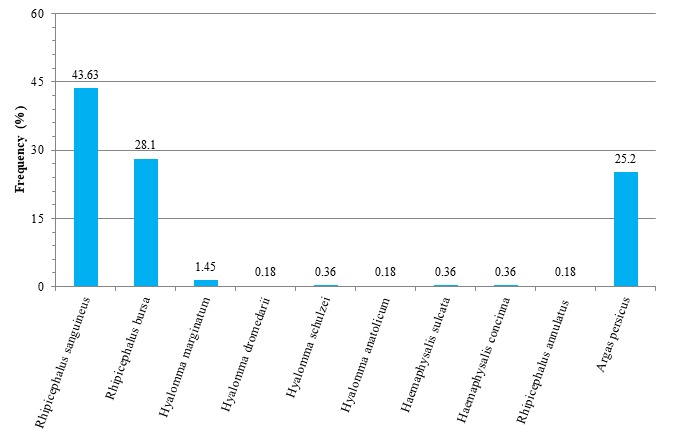
Frequency of tick species in the Alashtar county from April to March 2014

From the collected hard ticks, the male and female rates were 48.4% and 51.6%, respectively. There was no significant difference between the tick sex rates (P > 0.05). The tick distribution was more from plain areas (64.96%) than the mountainous areas (35.04%) (Table 4). Chi-square analysis showed a significant difference between the tick distribution in the plain and mountainous areas (P < 0.05). The most frequent species were *Rhipicephalus sanguineus* and *Rhipicephalus bursa* in the mountainous and plain areas while the other species had low abundance in the mentioned areas (Table 4). The rates of the tick contamination were 97.3% and 2.7% in the traditional and industrial livestock's, respectively (Table 4). Chi-square analysis showed a significant difference between the traditional and industrial livestock's tick contamination (P < 0.05). *Rhipicephalus* was observed as the most frequently genus in the traditional and industrial livestock's that their species were *Rhipicephalus sanguineus* and *Rhipicephalus bursa* while the other species had low abundance. All the soft ticks were collected from the traditional livestock's (Table 4). The most common identified hard tick species (Ixodidae) were *Rhipicephalus sanguineus* (43.63.1%) and *Rhipicephalus bursa* (28.1%) while the others were *Hyalomma marginatum*, *Hyalomma dromedarii*, *Hyalomma schulzei*, *Haemaphysalis sulcata*, *Rhipicephalus annulatus* and *Haemaphysalis concinna* with a very low frequency. All of the soft ticks (Argasidae) identified as *Argas persicus* species (25.2%) (Figure 4). The livestock contamination ranks to the hard ticks were cattle (39.51%), sheep (34.15%) and goats (26.34%), respectively. The *Rhipicephalus bursa* and *Rhipicephalus sanguineus* species were created the most contamination on the cattle and goats and sheep, respectively while the other species were less created livestock's contamination (Table 5).

**Table 4:** Frequency of the sex tick species and tick species distribution in the different rural and geographically location of the Alashtar county from April to March 2014

Species	No.	%	♂		♀		Plain		Mountainous		Traditional		Industrial	
			No.	%	No.	%	No.	%	No.	%	No.	%	No.	%
*Rhipicephalus sanguineus*	239	43.63	119	49.7	120	50.2	160	66.9	79	33.1	231	96.7	8	3.3
*Rhipicephalus bursa*	155	28.1	70	45.1	85	54.9	107	69.5	47	30.5	147	95.5	7	4.5
*Hyalomma marginatum*	8	1.45	8	100	0	0	2	25.0	6	5.0	8	100	0	0
*Hyalomma dromedarii*	1	0.18	1	100	0	0	0	0	1	100	1	100	0	0
*Hyalomma schulzei*	2	0.36	1	50	1	50	0	0	2	100	2	100	0	0
*Haemaphysalis sulcata*	2	0.36	0	0	2	100	0	0	2	100	2	100	0	0
*Hyalomma anatolicum*	1	0.18	0	0	1	100	0	0	1	100	1	100	0	0
*Rhipicephalus annulatus*	1	0.18	0	0	1	100	0	0	1	100	1	100	0	0
*Haemaphysalis concinna*	2	0.36	0	0	2	100	0	0	2	100	2	100	0	0
*Argas persicus*	138	25.2	-	-	-	-	87	63.0	51	37.0	138	100	0	0

**Table 5:** Livestock type contamination in the Alashtar county from April to March 2014

Species	Cattle		Sheep		Goat	
	No.	%	No.	%	No.	%
*Rhipicephalus sanguineus*	42	17.6	113	47.0	84	35.1
*Rhipicephalus bursa*	109	70.8	24	15.6	21	13.6
*Hyalomma marginatum*	7	87.5	1	12.5	0	0
*Hyalomma dromedarii*	1	100	0	0	0	0
*Hyalomma schulzei*	1	50	0	0	1	50
*Haemaphysalis sulcata*	1	50	0	0	1	50
*Hyalomma anatolicum*	0	0	1	100	0	0
*Rhipicephalus annulatus*	1	100	0	0	0	0
*Haemaphysalis concinna*	0	0	1	50	1	50

## Discussion

In this study, the three genera and nine species of the hard ticks (Ixodidae) and one genus and species of the soft ticks (Argasidae) were identified. The genera and species of the hard ticks were *Haemaphysalis*, *Hyalomma*and *Rhipicephalus* and *Haemaphysalis concinna* (0.36%), *H. sulcata* (0.36%), *Hyalomma anatolicum* (0.18%), *Hy. dromedarii* (0.18%), *Hy. marginatum* (1.45%), *Hy. schulzei* (0.36%), *Rhipicephalus annulatus* (0.18%), *R. bursa* (28.1%) and *R. sanguineus* (43.63%), respectively. The one genus and species of the soft ticks were *Argas* and *Argas persicus* (25.2%), respectively (Figure 4). A study conducted in the Bukan county (West Azerbaijan province) showed that the animal tick contamination rates were 88.57, 31.03 and 18.3 for goats, sheep and cattle, respectively. While in the present study the animal tick contamination rates were 26.34, 34.15 and 39.51 for goats, sheep and cattle, respectively (Table 5). As our study, *Haemaphysalis* and *Rhipicephalus*were the most frequent genera and *Rhipicephalus sanguineus* had the most contamination rates (85.7%) [19]. In a survey the species compositions of hard ticks of livestock in Boeen Zahra and Takistan counties of Qazvin province were studied. The species compositions from the livestock of Boeen Zahra were *H. concinna* (0.63%), *H. sulcata* (12.66%), *Hy. anatolicum* (3.80%), *Hy. Asiaticum* (3.16%), *Hy. detritum* (5.70%), *Hy. Dromedarii* (28.48%), *Hy. marginatum* (13.29%), *Hy. Schulzei* (1.89%), *Rhipicephalus bursa* (3.16%) and *R. sanguineus* (3.16%) and for Takistan's livestock were *Hy. dromedarii* (9.86%), *Hy. marginatum* (13.29%), *Hy. Schulzei* (1.89%) and *R. sanguineus* (3.16%), respectively [18]. Among our tick detected species, *Haemaphysalis concinna* distribute widely in the Palearctic and Oriental Zoogeographic regions such as China, Russia and Poland, as well as some parts of temperate Eurasia. *H. concinna* are found mainly in shrubs and grasslands habitats. Field study in north China has been showed that it attacks to domestic animals and humans, with heavy infestations occurring in summer [23, 24]. *H. concinna* has significant importance because it is a three-host tick vector of several pathogens of humans and livestock. It has been reported to carry pathogens including Lyme borreliosis spirochetes, Far-Eastern subtype of tick-borne encephalitis virus, *Coxiella burnetii*, *Rickettsia sibirica* and Crimean-Congo hemorrhagic fever virus (CCHFV) [24]. In the last decade, the Crimean-Congo hemorrhagic fever (CCHF) has become a growing public health concern and the most important viral tick-borne disease in Iran and Turkey. Between 2004 and 2013, a total of 390 deaths were reported in Turkey. During 1999-2011, 871 human cases of CCHF were also diagnosed in Iran [11, 25]. As previous studies (0.06 and 0.63%) [16, 18], present study shows that *H. concinna* is less commonly encountered (0.36%) and found in spring. In Iran, as present study which found in the mountainous areas of the Lorestan province (Table 3, Table 4, Figure 4) it is commonly consisted in the east of the Caspian sea zone to southern mountainous areas such as Guilan, Mazandaran and Golestan and Azarbaiejan, Ardebil, Kohgilouyeh and Kordistan, respectively. As it is collected on the sheep and goat confirm pioneer researchers that they state to be relatively common in sheep pasture regions. This tick was found infected with rickettsiae of spotted-fever group, but it is considered not an important vector. Examining the *H. concinna* ticks collected in Kazakhstan revealed *Anaplasma bovis* and *Rickettsia hulinii*. Laboratory studies showed that *H. concinna* experimentally able to transmit *Borrelia* in China. This tick was also found to be infected with the causative agents of tularemia [16].

Adults and immature stages of *Haemaphysalis sulcata*, another detected species of genus *Haemaphysalis*, are active during the cold season and summer, respectively. The major hosts of its adults are large mammals including cattle and sheep while reptiles and birds are its hosts of immature stages. The temperate mesomediterranean and submediterranean bioclimates such as Iran, Turkey and neighboring countries are often natural habitats of *H. sulcata*. It is widely distributed in India, southern USSR, and from southwestern Asia to the western Mediterranean area. This tick reported from humid and sub-humid zones on cattle and sheep in Tunisia. *H. sulcata* is an economically important tick species that it may transmit several pathogenic organisms belong to the genera *Babesia* and *Theileria* or *Anaplasma ovis* species, which causes ovine anaplasmosis [16, 26–28]. In Iran *H. sulcata*, is widely distributed and commonly found from northeast to southeast in semi-dessert zones. The larvae and nymphs feed on a variety of rodents and many small and large animals, respectively. Adults are usually infested larger animals, such as wild and domestic sheep, goats, cattle, horses and camel [16]. As *H. concinna* and previous study (0.6%) [16], *H. sulcata* recorded with low frequency (0.36%) in the mountainous areas in the spring while Shemshad et al (2012) reported 12.66% in Qazvin province. Ixodids from the *Hyalomma* genus, the second our detected genus are important pests of livestock with major medical and veterinary significance [29]. One of the most geographically widespread arboviruses is the zoonotic CCHFV and causes a severe hemorrhagic syndrome in humans. Ticks of the genus *Hyalomma* are the main vectors and reservoirs of the virus that it circulates in nature in a vertebrate-tick cycle [30, 31]. The first detected species of *Hyalomma* genus was *Hyalomma anatolicum* that have various habitats extending from central parts of the Sudan to North Africa, Southern Europe, the Middle East, Russia, China and India. The tick behavioural or morphogenetic diapause occurs in cold climates while it can multiply throughout the year in hot climates. *Hy. anatolicum* has two to three-host types depends on its feeding behavior. On large domestic animals, it has a typical three-host feeding cycle while on small hosts (unusual hosts) has two to three-host types. It can maintain a range of economically important transmissible infections to domestic animals and humans. *Theileria annulata* (tropical theileriosis in cattle), *Theileria lestoquardi* (malignant sheep theileriosis), *Theileria equi* (equine babesiosis), CCHFV (to humans) and *Babesia caballi* (cause of equine babesiosis) are the infections that it transmits transstadially and transovarially, respectively [32]. *Hy. anatolicum* reported as the most predominant tick animal infesting for cattle (25.32%), sheep (28.36%) and goats (28.45%) in the natural habitat of Sanandaj suburb [20] while we observed it as infesting sheep (0.18%) in the mountainous areas in the spring. *Hyalomma dromedarii*, the second detected species of *Hyalomma* genus, is common in the Mediterranean region steppe and desert climates and is widely distributed throughout North Africa, the Northern regions of West, Central and East Africa; Arabia, Asia Minor, the Middle East and Central and South Asia. *Hy. dromedarii* can behave as a one-, two- or three-host species but two-host is the most common in its life cycle. It represents nearly 90% of ticks infesting camels [33, 34].

In Iran, *Hy. dromedarii* is scattered almost all over, especially in the areas where camels occur as large herds. *Hy. dromedarii* ticks were found predominant tick species (61.9%) engorging on many camels grazing around the Persian Gulf with relative humidity nearly up to 90% [14]. In present study were accounted 0.18% of the adult male ticks on cattle in the mountainous areas in the spring. *Hyalomma marginatum*, the third detected species of *Hyalomma* genus, is widely distributed in Southern Europe, Asia, Near and Middle East. *Hy. marginatum* is the most important vector for CCHFV in southern Europe as well as parts of the Middle East and Central Asia [31]. *Hy. marginatum* is widely distributed in entire Iran. Like our study (1.45%), Nazifi et al (2012) was recorded *H. marginatum* (1.9%), while was found 12.5 % and 13.29% by Razmi et al. (2007) and Shemshad et al. (2012), respectively. Current study is reperesented that *Hy. marginatum* feeds mostly on cattle around the mountainous areas in the spring. *Hyalomma schulzei*, the forth detected species of *Hyalomma*genus, is especially common in the Saravan area of northeast Iran, near the Pakistan border and they are distributed in a narrow belt from Afghanistan to the western desert of Egypt [13]. It is a robust and rare Iranian *Hyalomma* species and is considered to be the species most closely associated with camels [35]. We accounted 0.36% on cattle and goat around the mountainous areas in the spring. In previous studies *Hy. schulzei* were observed in the semi-desert area of the central Iran and Boeen Zahra and Takistan counties of Qazvin province with 4.03% and 1.89% frequency, respectively [13, 18]. Another species that we found was *Rhipicephalus annulatus* formerly named as *Boophilus annulatus*. It is one of the most common ticks in cattle in Iran. *R. annulatus* is an important one-host tick in the Mediterranean regions including Iran. It can transmit the *Babesia bigemina*, *Babesia bovis* and *Anaplasma marginale* to cattle and have a high frequency in Northern Iran [10]. *Rhipicephalus annulatus* have been identified as vectors of babesiosis and anaplasmosis in Mazandaran Province and reported from ruminants. After very low level of synthetic pyrethroid insecticide resistance reported from India, its larvae has been resisted to pyrethroid in Iran [36] that is the situation is worsening and its risk increases. This tick has also been recorded in some regions of Europe such as Portugal, Spain, Italy and Greece; Americas such as Mexico; and in northern Africa [37]. *R. annulatus* ticks were reported from Mazandaran province with 35.77%, 49.0% and 51.3% frequency [12, 17]. We accounted 0.^18^% on cattle around the mountainous areas in the spring. *Rhipicephalus bursa*, the second detected species of *Rhipicephalus* genus, occurs in Southern Europe, Near and Middle East. It has an important role to transfer agent of diseases such as *Anaplasma marginale*, *Babesia bigeminum*, *Babesia motasi* and *Babesia ovis* to animals [38]. *Ehrlichia canis*detected from R. bursa ticks in Italy [39]. *R. bursa* were mostly contaminated the sheep (90.7%) and goats (88.8%), followed by *R. sanguineus* (6.9%) and *R. annulatus* (2.4%) in Urmia suburb [2]. Totally *R. bursa* ticks were accounted 28.1% in current research with more frequency on cattle (70.8%) than sheep (15.6%) and goat (13.6%) and also more in the plain areas (69.5%) in the spring (61.7%) than the mountainous areas (30.5%) and other seasons. It was recorded 16.8% and 3.16% in Mazandaran and Qazvin provinces, respectively [17, 18].

The last detected species of *Rhipicephalus* genus was *Rhipicephalus sanguineus*, the brown dog tick. It is a three-host tick that infests dogs and occasionally can feed on a wide range of domestic and wild hosts, including cats, rodents, birds and humans. It widely distributed in the world and recognized vector of many pathogens, such as Babesia canis, *Ehrlichia canis*, *Coxiella burnetii*, *Rickettsia conorii* and *R. rickettsii* affecting dogs and occasionally humans affecting dogs and occasionally humans [40, 41]. In Iran it recorded as 34.8% and 3.16% in Sistan and Qazvin province, respectively [18]. As a total *R. sanguineus* ticks were observed 43.63%, the most frequent tick species, in present study with more frequency on sheep (47.0%) and goat (35.1%) than cattle (17.6%) and also more in the plain areas (66.9%) in the spring (56.9%) than the mountainous areas (33.1%) and summer (30.5%) autumn (7.1%) and winter (5.4%). Soft ticks can be a serious pest in poultry and pig operations in tropical and subtropical countries. Blood loss and subsequent anemia can be significant and substantially affect weight gains and egg laying performance. Massive infestations can cause numerous fatalities. *Argas persicus* is distributed worldwide in warm climates. In western parts of its palearctic areal this tick is known in the Middle East, Egypt, Libya and Maghreb on the Southern Mediterranean side and Anatolia, the Balkans (reaching Slovakia in the North and Trieste, Italy, in the West), Corsica and Spain in the North. It considered native in Turanian-Central Asia as a parasite of arboreal nesting birds and has successfully adapted to coexistence with domestic fowl. Probably via human transport, it has spread throughout the continents, where it survives practically exclusively in association with domestic fowl (chickens, turkeys, Helmeted Guinea fowls, and others) and infrequently pigeons. Heavy infections with *Argas spp.* can cause blood loss leading to anemia and eventually death. Also, *A. persicus* Larvae have been responsible for synchronous occurrence of infectious bursal disease and spirochaetosis. *A. persicus* infestation caused paralysis in infected birds. In some countries this tick is the most important poultry ectoparasite. *Argas* and *Ornithodorus* ticks are vectors of numerous poultry diseases such as *Borrelia anserina* (avian spirochetosis agent) and *Aegyptianella pullorum*(aegypitianellosis agent). *Argas* ticks also transmit *Pasteurella multocida* (fowl cholera agent) [15, 25, 42]. In china, identified as vector of *Borrelia anserine* (Avian spirochetosis), Kyasanur Forest disease virus (Kyasanur forest disease) and *Wolbachia persica* (Paralysis) [43]. In Iran *A*. *persicus* reported from Western Azerbaijan, Lorestan, and Khorasan Razavy provinces [21, 44], and Sistan region [15]. *A. persicus* ticks were collected 16.94 % in Sistan region [15] while we recored 25.2 % in our study. *A. persicus* ticks were observed with more frequency in the plain areas (63.0 %) in the spring (47.26 %) than the mountainous areas (37.0 %), and summer (22.63 %) autumn (14.96 %) and winter (15.15 %). Finally present study shows that the rates of the tick contamination were mostly recorded in the traditional livestock's (97.3 %) than industrial livestock's (2.7 %) (Table 4) that is proved to change the traditional livestock's to the industrial livestock's. Although a variety of disease agents can be currently spread by ticks, while it is possible to determine tick transmitting ability of the disease agents that they are not capable to transmit them by artificially contamination of ticks [45, 46].

## Conclusion

In this study, we identified ten tick species including nine hard (Ixodidae) and one soft tick species (Argasidae). The hard tick species were *Haemaphysalis concinna* (0.36%), *H. sulcata* (0.36%), *Hyalomma anatolicum* (0.18%), *Hy. Dromedarii* (0.18%), *Hy. marginatum* (1.45%), *Hy. schulzei* (0.36%), *Rhipicephalus annulatus* (0.18%), *R. bursa* (28.1%) and *R. sanguineus* (43.63%). These tick species that we found mostly have more than one host species. Some are able to transmit very important diseases such as ovine anaplasmosis, Crimean-Congo hemorrhagic fever. One of the most geographically widespread arboviruses is the zoonotic CCHFV and causes a severe hemorrhagic syndrome in humans. Ticks of the genus *Hyalomma* are the main vectors and reservoirs of the virus that it circulates in nature in a vertebrate-tick cycle. Some of these ticks can transmit *Theileria annulata* (tropical theileriosis in cattle), *Theileria lestoquardi* (malignant sheep theileriosis), *Theileria equi* (equine babesiosis), CCHFV (to humans) and *Babesia caballi* (cause of equine babesiosis) are the infections that it transmits transstadially and transovarially, respectively. Some of these ticks also can transmit the *Babesia bigemina*, *Babesia bovis* and *Anaplasma marginale* to animals and *Babesia canis*, *Ehrlichia canis*, *Coxiella burnetii*, *Rickettsia conorii* and *R. rickettsii* to dogs and occasionally humans. The soft tick species were *Argas persicus* (25.2%). Heavy infections with *Argas spp.* can cause blood loss leading to anemia and eventually death. *A. persicus* can transmit avian spirochetosis and aegypitianellosis agents. In china, *A. persicus* also identified as vectors of avian spirochetosis and Kyasanur forest disease agents. Finally present study shows that the rates of the tick contamination were mostly recorded in the traditional livestock's (97.3%) than industrial livestock's (2.7%), that is proves to change the traditional livestock's to the industrial livestock's. These findings highlight the importance of ticks and shows need to their control and tick pest management.

### What is known about this topic

- Ticks are non-permanent obligate and the most frequent ectoparasites (ticks, mites, lice and fleas) of the terrestrial vertebrates that have considerable medical-veterinary and zoonosis importance;
- They are a serious threat to animal health and public health in many parts of the world because they are able to direct damages and transmission of parasitic, viral and bacterial pathogens.

### What this study adds

- Ten tick species including *Haemaphysalis concinna* (0.36%), *Haemaphysalis sulcata* (0.36%), *Hyalomma anatolicum* (0.18%), *Hyalomma dromedarii* (0.18%), *Hyalomma marginatum* (1.45%), *Hyalomma schulzei* (0.36%), *Rhipicephalus annulatus* (0.18%), *Rhipicephalus bursa* (28.1%), *Rhipicephalus sanguineus* (43.63%) and Argas persicus (25.2%) were identified.
- Tick seasonal distribution were 47.26%, 22.63%, 14.96% and 15.15% in the spring, summer, autumn and winter, respectively. The tick distribution was more from plain areas (64.96%) than the mountainous areas (35.04%). The rates of the tick contamination were 97.3% and 2.7% in the traditional and industrial livestock's, respectively. The livestock contamination ranks to the hard ticks were cattle (39.51%), sheep (34.15%) and goats (26.34%), respectively.

## Competing interests

The authors declare no competing interest.
